# Synergistic Effect of Fluorinated and N Doped TiO_2_ Nanoparticles Leading to Different Microstructure and Enhanced Photocatalytic Bacterial Inactivation

**DOI:** 10.3390/nano7110391

**Published:** 2017-11-15

**Authors:** Irena Milosevic, Amarnath Jayaprakash, Brigitte Greenwood, Birgit van Driel, Sami Rtimi, Paul Bowen

**Affiliations:** 1 Ecole Polytechnique Fédérale de Lausanne, EPFL-STI-IMX-LTP, Station 12, CH-1015 Lausanne, Switzerland; amarnath.jayaprakash@epfl.ch (A.J.); brigitte.greenwood@gmail.com (B.G.); b.a.vandriel@tudelft.nl (B.v.D.); sami.rtimi@epfl.ch (S.R.); paul.bowen@epfl.ch (P.B.); 2 Ecole Polytechnique Fédérale de Lausanne, EPFL-SB-ISIC-GPAO, Station 6, CH-1015 Lausanne, Switzerland

**Keywords:** TiO_2_, N doping, fluorination, wet milling, heat treatment, synergy, disinfection

## Abstract

This work focuses on the development of a facile and scalable wet milling method followed by heat treatment to prepare fluorinated and/or N-doped TiO_2_ nanopowders with improved photocatalytic properties under visible light. The structural and electronic properties of doped particles were investigated by various techniques. The successful doping of TiO_2_ was confirmed by X-ray photoelectron spectroscopy (XPS), and the atoms appeared to be mainly located in interstitial positions for N whereas the fluorination is located at the TiO_2_ surface. The formation of intragap states was found to be responsible for the band gap narrowing leading to the faster bacterial inactivation dynamics observed for the fluorinated and N doped TiO_2_ particles compared to N-doped TiO_2_. This was attributed to a synergistic effect. The results presented in this study confirmed the suitability of the preparation approach for the large-scale production of cost-efficient doped TiO_2_ for effective bacterial inactivation.

## 1. Introduction

Nowadays, advanced oxidation processes (AOPs) for disinfecting water are drawing more attention [1,2]. AOPs are chemical treatment procedures that remove organic pollutants by oxidation through reactive oxygen species (ROS). Hydroxyl radicals (•OH) are one of the most oxidative ROS, with a potential of 2.80 V vs. normal hydrogen electrode (NHE) [3]. Photocatalysis refers to the process of using light to accelerate the kinetics of a chemical reaction while remaining unconsumed. Upon irradiation with light, when the energy of an excitation source is greater than the band gap energy of the photocatalyst, photon absorption occurs. The latter leads to the excitation of an electron (e^−^) from valence to conduction band leaving behind an electronic hole (h^+^). These photogenerated pairs (electron/hole) can now undergo recombination or create ROS [4]. The charge carriers can migrate to the surface of the photocatalyst to initiate reactions with surface adsorbed molecules (mainly H_2_O and/or O_2_). The photoexcited electron can react with oxygen to form superoxide radicals (•O_2_^−^) and the holes react with water to form hydroperoxide radicals (HO_2_•). The ROS can attack the bacterial cell wall, eventually causing cell wall disruption leading to cell death [5]. 

Ever since TiO_2_ photocatalysis was observed by Fujishima and Honda [6,7], TiO_2_ has been widely used for photocatalysis. TiO_2_ is cheap, inert, chemically stable and environment friendly. Photocatalysis with TiO_2_ can be achieved at ambient temperature and pressure. However, the major drawback of TiO_2_ in photocatalysis is its relatively large band gap. This large band gap restricts light absorption to only ultra violet (UV) light and induces the fast recombination of photoinduced electrons and holes, which restricts the improvement of solar energy conversion efficiency. Since the UV spectrum is only a minute portion of the light reaching the earth’s surface [8], TiO_2_ is ineffective for indoor and/or confined environments. Doping TiO_2_ can create color centers that lead to visible light absorption [9] and enable its activity under visible light. Many researchers have proved the possibility of tuning the band gap resulting in the activation of TiO_2_ under visible light by doping with metallic [10,11] and non-metallic [12] elements, using either a self-doping process [13] or by deposition of noble metals on their surface [14,15]. N-doped TiO_2_ is one of the most promising and studied anion-doped TiO_2_ for photocatalytic applications. Several methods were used to dope TiO_2_ such as sol-gel synthesis [16,17], sputtering in N_2_ [18,19], ion implantation [20,21], gas phase reaction [22,23], and atomic layer deposition [24], as well as dry and wet milling followed by heat treatment [25]. 

In this study, we choose to work with P25 as a source of TiO_2_, which is a commercial nanopowder. P25 is a mixture of 70% anatase and 30% rutile phases. It was shown that this biphasic TiO_2_ showed more superior photocatalytic activities than pure-phase TiO_2_, owing to the efficient electron transfer from the conduction band of the anatase to that of the rutile phase [13,26]. 

A wet milling method of TiO_2_ in the presence of a doping agent with a suitable solvent is a simple scalable method that has been shown to be effective in previous studies [27]. The wet milling method has advantages such as reduced agglomeration over dry milling [28], which showed signs of heavy agglomeration.

Senna et al. [27] showed that the doping of TiO_2_ with nitrogen through wet milling and subsequent annealing gave catalytic activity in indoor light by modifying the absorption spectrum. TiO_2_ doped with both fluorine and nitrogen showed better photoabsorption of the visible spectrum than doping with either [29]. Moreover, doping TiO_2_ with fluorine and a combination of fluorine and nitrogen through wet milling has not been reported before. Thus, the present work studies each sample individually, by spectroscopy methods, powder granulometry and electron microscopy, and tests their visible light absorption and antibacterial activity taking *E. coli* as a probe. 

## 2. Results

### 2.1. Untreated Particles and Effect of Attrition Milling 

The adsorption of the dopant molecules is a crucial step in our process, as their vicinity will further help the doping by the direct diffusion of ions from the surface into the TiO_2_ structure [27]. By following the surface potential changes, the adsorption of the molecules on P25 and the consequent surface modification after heat treatment can be observed. Table 1 presents the zeta potential values of attrition milled and heat-treated samples. The negative zeta potential values are in good agreement with results showing the isoelectric point around pH 5–6 for P25 [5], which as stated is not altered so much by the ionic strength of the culture medium (not presented in the table). We notice that after the attrition process, samples P25-N-Att, P25-F-Att, and P25-F&N-Att have a much lower zeta potential absolute value compared with P25-UN, which is probably due to the successful adsorption of the doping molecule (Glycine (Gly) and/or polytetrafluoroethylene (PTFE)) onto the surface of the nanoparticles (NPs) by wet milling. It is worth noting that PTFE has a poor solubility in water; thus, attrition mill seems to be an ideal technique in this case.

After heat treatment (HT), all of the “doped” samples have a clearly negative surface potential charge. Indeed, doped P25 samples showed an increase in their zeta potential absolute value after the burning of the organic layer at high temperature evidencing a surface change consequent to the doping process. 

Due to the negative zeta potential of the doped NPs, electrostatic repulsion is to be expected between bacterial cell surfaces and NPs at this working pH, since both are negatively charged. Yet, toxicity is still observed, as later discussed in the section on the antibacterial trials. We used the thermogravimetric analysis (TGA) to study the weight loss of organic species of the attrition milled samples during calcination.

**Figure 1:**
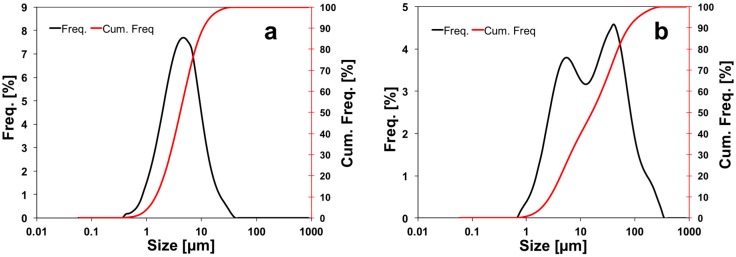
Laser diffraction (Malvern) particle size distribution of (**a**) P25-UN and (**b**) P25-F&N-HT.

A weight loss was registered for all the samples from 200 °C to 500 °C or 600 °C. The TGA results of the N-attrited P25 (Figure 1) show that 3 wt % of Gly was deposited on the surface of the P25 sample after attrition milling. For P25-F-Att and P25-F&N-Att, we observed a higher weight loss of 9 wt %, which was close to the 10% of PTFE added when compared with the dry TiO_2_ powder (see Supplementary, Figure S1).

### 2.2. Experimental Change upon HT

The particle size distributions (PSD) that were measured using laser diffraction for the parent powders after attrition milling, drying, and heat treatment are given in Table 1. The samples were prepared in the saline buffer solution (SBS) medium used for the antibacterial test followed by magnetic stirring to characterize in the same experimental conditions. PSDs and morphological changes over the course of treatment are evaluated, and possible explanations and correlations with other results are now discussed. 

Figure 1 shows a typical Malvern measurement of our sample, revealing in all cases micrometer size agglomerates. The PSD of P25-UN dispersed by means of ultrasounds in 1 mM HNO_3_ gave a single mode at 2.43 μm. These values shifted towards bigger sizes, up to 3.28 μm, by dispersing the particles using magnetic stirring in the SBS, as shown in Table 1. The ionic strength of the SBS, in addition to the lack of a high-energy dispersing step, is probably playing an important role on the aggregation state of our samples. Indeed, salt-induced aggregation is a well-known process in particle suspensions [30].

After the doping process involving HT at either 500 °C or 600 °C, D_v_50 increases with aggregates five to seven times bigger than the P25-UN. The span of Malvern D_v_50 is two times higher than the parent D_v_50, showing that the heat treatment has a significant impact on the average particle size and consistently widens the size distribution. This suggests that some of the agglomerates began to sinter and/or transform into rutile [31] during the heat treatment [32]. 

**Figure 2:**
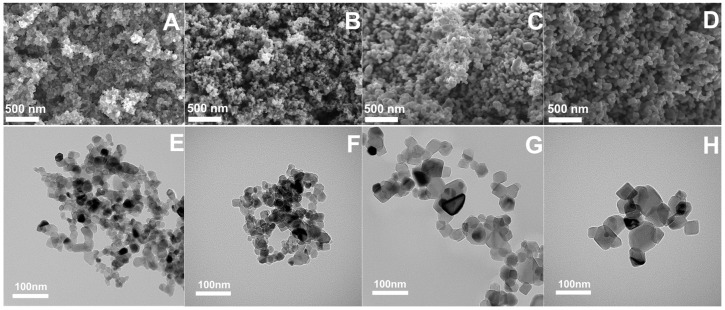
SEM and TEM images respectively of (**A**,**E**) untreated P25; (**B**,**F**) P25-N-HT; (**C**,**G**) P25-F-HT; and (**D**,**H**) P25-F&N-HT.

The SEM images in Figure 2 show little morphological change due to the HT process, but in every case, heavy agglomeration can be noticed. In TEM micrographs, bigger and more well-define particles are clearly observed when the heat treatment is done at 600 °C (Figure 2G,H) compared with P25-N-HT treated at 500 °C (Figure 2F). The change can be also correlated to the partial sintering and/or transformation into rutile of P25 particles at high temperature with respect to the untreated TiO_2_ (Figure 2E).

The Brunauer-Emmett-Teller (BET)—specific surface area (SSA) in Table 1 shows an increase after attrition milling heat treatment for P25-N-HT. This is further clarified by calculating the primary particle diameter obtained from BET, d_BET_, and the correlated agglomeration factor, F_ag_, from Equations (1) and (2) respectively [33]:

(1) $$d_{\mathrm{BET}}=6÷\left(\mathrm{SSA}\times \rho \right)$$

(2) $$F_{\mathrm{ag}}=D_{v}50\left(\mathrm{Malvern}\right)÷d_{\mathrm{BET}}$$

where ρ is the density of the specimen, and D_v_50 is the volume median diameter of the particle size distribution.

As the SSA increases, the d_BET_ decreases for P25-N-HT, as expected, from 35 nm (P25-UN) to 28 nm (P25-N-HT). This additionally proves that there is no significant partial sintering observed at 500 °C as confirmed by SEM and TEM (Figure 2B,F). Fluorinated samples however, show a decrease in SSA coupled with an increase in d_BET_ to twice the size of P25-UN, which relates to the partial sintering in P25-F-HT and P25-F&N-HT resulting from heat treatment at 600 °C. 

After HT, the particles appeared more agglomerated in all cases after dispersion in SBS. Nevertheless, we can notice an agglomeration factor for P25-N-HT that is more than two times higher than of both P25-F-HT and P25-F&N-HT, whereas almost no difference is observed between the latter two. Moreover, it is worth noting that the F_ag_ is strongly dependent on the dispersing medium and dispersing technique. Indeed, by suspending the P25 nanoparticles in HNO_3_ solution and applying additional ultrasound technique to help break the agglomerates, the F_ag_ is found to be 69 compared to 94 in SBS for the same sample. 

The chemical composition of the as-doped sample surfaces was investigated by X-ray photoelectron spectroscopy (XPS) analysis. The atomic concentration of the dopants in P25 were estimated to be, (i) P25-N-HT: around 0.30 at% of N, (ii) P25-F-HT: around 0.45 at% of F, (iii) P25-F&N-HT: around 0.45 at% of N and 1.00 at% of F, showing the successful doping of P25 through wet milling process using Gly and/or PTFE molecules.

**Figure 3:**
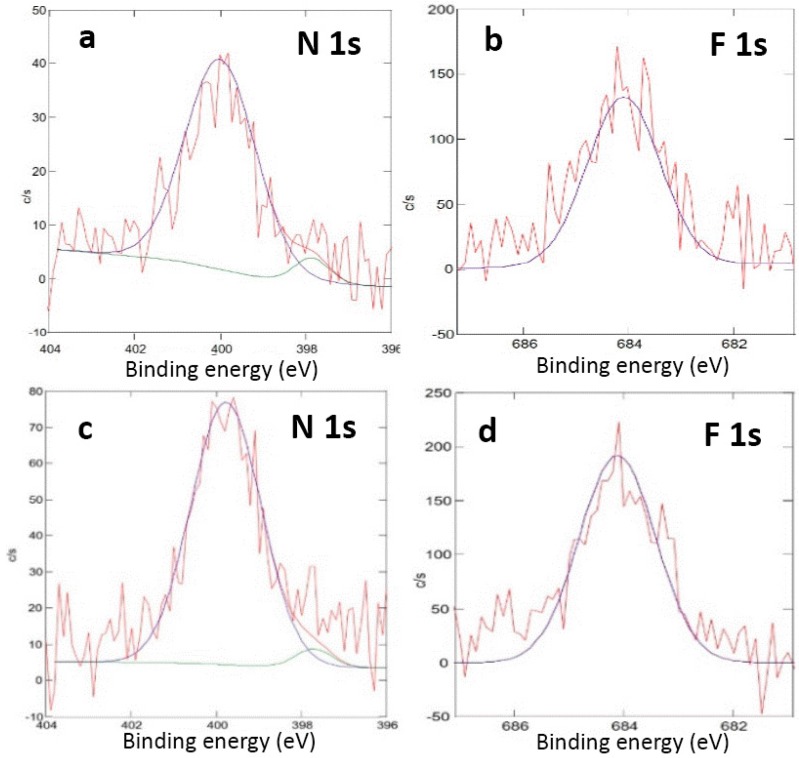
High-resolution X-ray photoelectron spectroscopy (XPS) spectra analysis in the N 1s and F 1s binding energy region for (**a**) P25-N-HT, (**b**) P25-F-HT, (**c**,**d**) P25-F&N-HT. All the other spectra are presented in SI, Figure S2.

For a detailed analysis, the curve fitting and deconvolution spectra of the Ti 2p, O 1s regions are given in Supplementary, Figure S2; which the N 1s and F 1s regions in the XPS spectra of doped samples are presented in Figure 3. The XPS spectra of Ti 2p revealed two main peaks that can be deconvoluted. The consequent binding energies (BE) could be assigned to Ti^3+^ at 457.8 eV and 463.1 eV and Ti^4+^ at 458.4 eV and 464.1 eV. The coexistence of the Ti^3+^/Ti^4+^ redox couple is crucial for photocatalysis applications [34]. In our case, the intensity of the peaks corresponding to Ti^3+^ is very low. The deconvolution of the O 1s peak in the XPS spectrum brought to light two peaks, at about 529.5 and 531.5 eV. The first one which is the main peak is ascribed to the O_2_ bonded to Ti in the form of Ti–O linkages [35], and the second peak of lower intensity can be attributed to the presence of OH groups [36]. 

The presence of one N 1s peak at BE from 396 eV to 398 eV is generally assigned to the substitutional doping site (Ti–N–Ti species) [20,37,38]. Samples doped with N and doped with both N and F showed a N 1s peak in the region between 392 and 406 eV (Ti–N–O) that can be deconvoluted into two bands with two different BE at 400 eV and 397.9 eV, revealing two kinds of nitrogen linkages in our catalysts. The highest intensity peak at 400 eV relates to interstitial doping, as stated by different groups [38,39,40,41]. In addition, this higher binding energy, which was confirmed more recently by Batalović et al. using Density functional theory (DFT) calculations, is attributed to interstitial nitrogen located near the lattice oxygen forming a short N–O bond [42]. The peak at 397.9 eV, despite a poor signal-to-noise ratio and low intensity, can possibly be assigned to atomic nitrogen bonded to titanium, with the N atom replacing the oxygen atoms in the TiO_2_ crystal lattice to form a N–Ti–N bond [43]. However, this is only representative of <5% of the N 1s signal. No signal for the nitrogen species was observed for undoped and F-doped sample.

The XPS spectra in the F 1s region for both the F and F&N modified samples are given in Figure 3B,D, respectively. A single peak is observed; the peak maximum is at 684.5 eV. This peak is attributed to the fluorine anions adsorbed at the surface of the nanoparticles (surface fluorination, i.e., to a terminal Ti–O–F or Ti–F bond), if we compare with literature data [44]. Furthermore, Senna et al., whom worked also with PTFE as a source of fluorine ion, attributed the peak at 684 eV to a change in electronic states due to fluorine incorporated into P25 near the surface region [28]. No evidence of a resolved peak in the region of 688 eV, which was assigned to substitutional lattice fluorine ions [44], was obtained in our XPS investigations. In our process using PTFE, the presence of this peak would not be a proof of the substitutional doping, since this peak is also found in pristine PTFE [28]. As expected, fluorine species were not detected in undoped and N-doped samples. 

**Figure 4:**
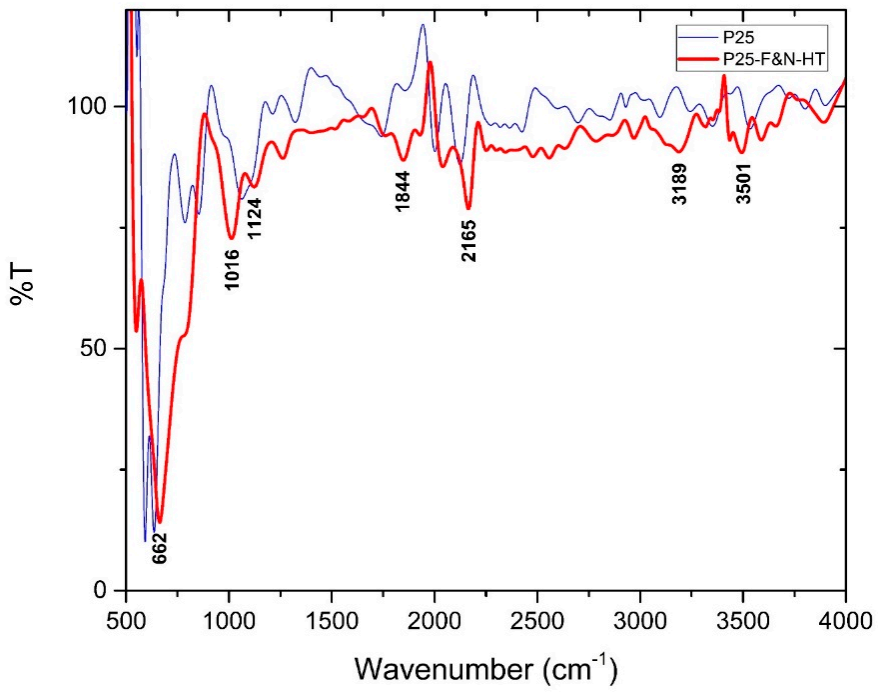
Fourier transform infrared (FTIR) spectra of P25-UN and P25-F&N-HT samples.

Fourier transform infrared (FTIR spectra of all of the samples have been investigated. All of the doped samples present similar features by FTIR. For clarity, only spectra of P25-UN and P25-F&N-HT are shown in Figure 4. The band between 500–800 cm^−1^ corresponds to the symmetric stretching vibrations of Ti–O and Ti–O–Ti [45,46]. The peak found at 1016 cm^−1^ is assigned to a Ti–O–C bond. Pillai et al. have correlated this bond with urea concentrations in their samples. Hence, it can be assumed that these peaks could be due to the carbon residue remaining on the surface of NPs after HT. The peak obtained at 1124 cm^−1^ is assigned to the stretching vibration of C–N [47]. The band at 2165 cm^−1^ is expected to be due to NO vibration [38]. The peak at 3189 cm^−1^ is characteristic of C–O stretching mode [48]. The two peaks located at 3501 and 1844 cm^−1^ represent the stretching [47] and bending vibrations [46] of the hydroxyl group on the surface of the powder and O–H bending of dissociated or molecularly adsorbed water molecules [45]. As shown in Figure 4 it is clear that the magnitude of the spectrum (at 3501 and 1844 cm^−1^) of doped samples is higher than for the untreated parent powder. This difference indicates higher surface adsorbed water and hydroxyl groups, which may play an important role in photocatalytic reactions [45,49]. 

**Figure 5:**
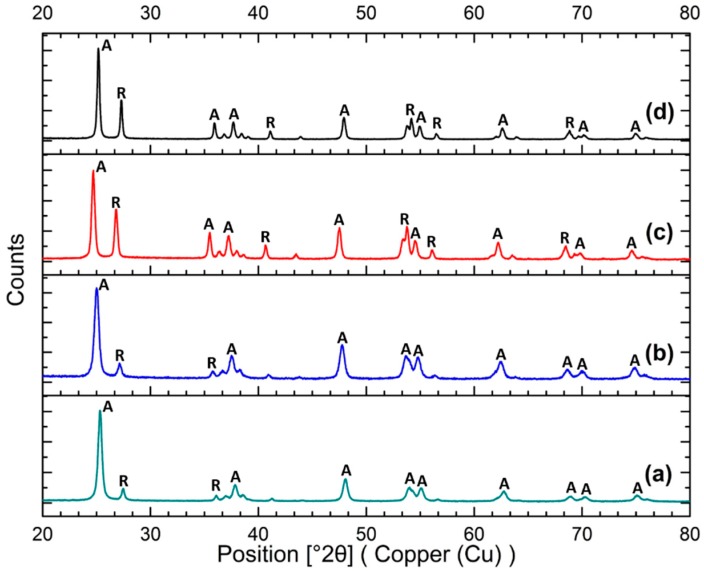
XRD spectra indexed with rutile (R) phase (RRUFF R110109) and anatase (A) phase (RRUFF R060277) of (**a**) P25-UN, (**b**) P25-N-HT, (**c**) P25-F-HT, (**d**) P25-F&N-HT.

The possible changes in the phase structure of the doped samples after HT were investigated by X-Ray Diffraction (XRD). Figure 5 shows the XRD patterns of P25 powders as-received and doped with N, Fluorinated, or doped with both N and F. All of the samples were analyzed along with a 100% rutile standard, which was used to quantify amorphous phases present (if any) after heat treatment of the samples. The analyses showed only crystalline powders with no traces of amorphous phases. In all of the samples, the indexation confirmed the presence of both anatase (RRUFF R060277) and rutile (RRUFF R110109) crystal structures with a dominant anatase phase. 

The P25-UN nanopowder showed around 90% anatase and 10% rutile (expected values from the Degussa data sheet were 70% anatase and 30% rutile). Almost the same proportion was found in P25-N-HT, whereas for both P25-F-HT and P25-F&N-HT, a significant change in anatase and rutile proportions were observed with almost 60% of anatase and 40% of rutile. The usual temperature of the phase transition from anatase to rutile TiO_2_ is around 550 °C [50]; thus, it can be concluded that the nanomaterial is gradually converted to rutile over this temperature range.

In the case of nitrogen doping, no change in the peak positions were noticed compared with P25-UN powder. Fluorine doping did not cause any shift in the XRD peak positions of P25-F-HT or P25-F&N-HT nanoparticles, which is in accordance with the XPS results showing no incorporation of fluorine into the particle. Moreover, since the radius of fluorine anion (0.133 nm) is almost the same as oxide anion (0.132 nm), no change in XRD pattern should even be observed in the case of substitutional doping.

The structure of the P25 samples was further investigated by Raman spectroscopy. The Raman spectra are shown in Supplementary, Figure S3. The spectra of P25-UN and P25-N-HT are comprised of four Raman modes at 138, 392, 514, and 635 (±3) cm^−1^ corresponding to E_g_(1), B_1g_(1), B_1g_(2) + A_1g_, and the E_g_(2) Raman active modes of the anatase phase. No other phase is observed in these spectra whereas the spectra of P25-F-HT and P25-F&N-HT revealed two more peaks at 444 and 606 cm^−1^, which are attributed to rutile phase E_g_ and A_1g_ modes along with a shift in anatase peaks [51,52]. The observation of the rutile phase here is due to its higher content as shown by the XRD results.

### 2.3. Photocatalytic Activity

Reflectance spectroscopy (RS) measurements were performed on all of the samples. The absorption of the samples is plotted in Supplementary, Figure S4 in the absorbance units vs. wavelength. Compared with parent P25, RS showed successful light absorption in the visible region (380–420 nm) for all of the doped samples, and more especially, fluorination influenced the light absorption characteristics. 

Our N-doped samples were prepared with a similar protocol, but with lower milling times and lower glycine concentrations than in the process of Senna et al. [27]. Here, our N-doped P25 samples showed better absorption compared with the P25-UN showing that N doping gives successful visible light absorption. Similar to other studies in the literature, we observed a better light absorption in the visible range for our N-doped [27,42] and F-doped samples [28]. Moreover, P25-F-HT and P25-F&N-HT showed a similar trend for the same anatase-to-rutile ratio. All of these results showed that doped samples changed the electronic structures of P25 that were associated with lower band gap energy. Similar modifications in the adsorption spectra have been previously observed for N and F monodoped anatase nanoparticles [42,53]. However, it was found that F&N modification considerably enhanced the visible light absorption of TiO_2_ as observed in our experiments [34,54].

**Figure 6:**
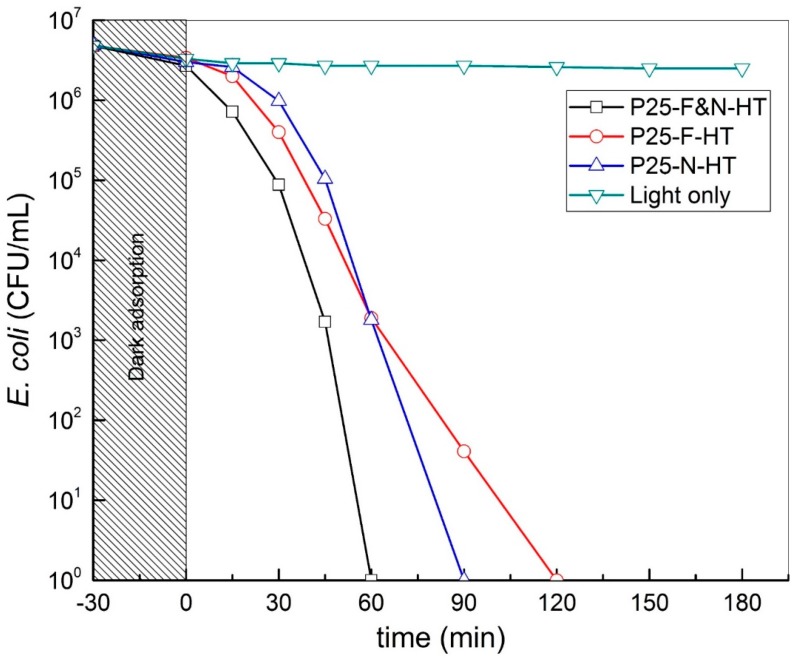
*E. coli* survival versus time in the presence of (1) light only or doped samples (2) P25-N-HT, (3) P25-F-HT, and (4) P25-N&F-HT under simulated light.

The photocatalytic performance of the prepared catalysts was tested to disinfect *E. coli* bacteria in suspension, as shown in Figure 6. All of the samples showed effective photocatalytic activity against *E. coli* leading to bacterial inactivation under simulated light. The most effective one was the F&N sample, which presented the fastest inactivation dynamics of *E. coli* compared with N and F monodoped TiO_2_.

## 3. Discussion

Photocatalytic activity was observed for doped samples, suggesting the possibility of creating intermediate energy levels in the band gap. Then, the production of ROS is possible under light irradiation, as it showed a soft spectral shift towards visible wavelengths, leading to the bacterial increase in the cell wall fluidity and the bacterial inactivation [55]. This agrees with the observed modification in the FTIR spectra showing higher surface adsorbed water and hydroxyl groups for doped samples. Indeed, as seen in Equations (4) and (5), (i) hydroxyl groups capture photoinduced holes (h^+^) when irradiated with light, and form hydroxyl radicals (•OH) with high oxidation capability [45], and (ii) surface adsorbed water acts as absorption centres for O_2_ molecules and form •OH to enhance photocatalytic activity [56].

H_2_O + vb(h^+^) → •OH + H^+^ (3)

O_2_ + cb(e-) → O_2_•^−^ (4)

At the molecular level, the photogenerated electrons tend to reduce Ti^4+^ to Ti^3+^, and the holes react with the bridging oxygen sites leading to oxygen vacancies and free •OH radicals. Redox couple Ti^3+^/Ti^4+^ were seen to co-exist in our prepared catalysts, as shown with XPS in SI, Figure S5. Water molecules heal the oxygen vacancies, producing OH groups on the surface, which leads to the oxidation of Ti^3+^ into Ti^4+^, as recently reported [56].

**Scheme 1:**
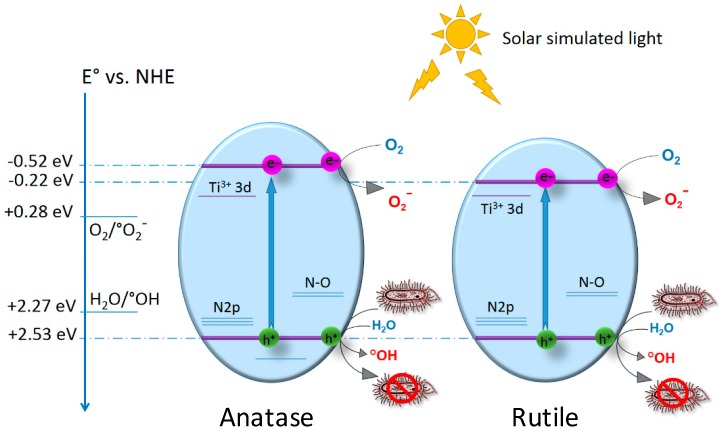
Schematic representation of the electron transfer mechanism showing the energy states and the role of various reactive oxygen species during the photocatalytic processes in the N- and F-doped anatase and rutile phase. P25 nanoparticles are a mixture of both anatase and rutile phases.

Substitution can occur during the heat treatment process, according to the atomic radii of the species. In our case, the atomic radius of N is 0.56 Å, which cannot substitute the Ti presenting a radius of 1.76 Å; however, it can substitute the O (0.48 Å), as recently reported [9,45,56]. This can lead to lattice (or interstitial) or O atom substitutional doping by N, which leads to intragap states and spectral absorptio. These Ti–N, O–N and Ti–O–N bonds were previously described in detail in the XPS section. For fluorinated & N doped TiO_2_, it has been demonstrated that the position of N (interstitial or substitutional) does not qualitatively differ in the essential features of the electronic structure [57]. Moreover, the important role of surface fluoride was highlighted in a N-doped TiO_2_ sample [58]. Scheme 1 shows the suggested mechanistic considerations behind the observed photocatalytic activity of the doped TiO_2_.

The N-doped catalyst showed no activity in the dark (Figure S5) and faster bacterial inactivation kinetics within 90 min compared with the P25-F-HT sample (120 min). The fluorinated & N cdoped sample presented the fastest inactivation dynamics of *E. coli*, with a total bacterial inactivation within 60 min. These results tend to prove that the antibacterial activity is not heavily size dependant, since similar aggregates size in both P25-F-HT and P25-F&N-HT lead to different results. Moreover, the partial sintering process for the fluorinated and fluorinated and N doped samples (at 600 °C) does not appear to affect their photocatalytic activities. This is in accordance with other studies that showing that NPs agglomerate—and not single NPs—interact primarily with *E. coli* cell walls before disrupting them [55].

Another point was to confirm whether the anatase/rutile ratio plays a role in the process. Indeed, we observed a phase transition for the samples HT at 600 °C, but for the same ratio in the fluorinated samples, we found different bacterial inactivation times (by a factor of two), meaning that the proportion of rutile is not crucial at this stage. Finally, these results show that a combination of both N and F lead to better activity against *E. coli* than fluorine or nitrogen separately. 

Fluorinated and N doped TiO_2_ (F&N-TiO_2_) demonstrated the fastest bacterial inactivation under solar light (UV and visible). Indeed, the presence of fluorine increases the catalytic activity of F&N-TiO_2_ when compared with N monodoped TiO_2_ [57]. Compared with the undoped TiO_2_, F&N-TiO_2_ showed improved activity under visible light. The slight improvement in the photocatalytic activity of the fluorinated TiO_2_ can be attributed to the enhanced intrinsic properties of UV photons absorption. Poor activity is observed in the visible light region (data not shown) due to the insufficient absorption in the lower energy range. In both N-TiO_2_ and F&N-TiO_2_, enhanced photocatalytic activity is observed in the visible region. This can be attributed to the band gap tuning which triggers photons absorption in the visible light range (Scheme 1). Consequently, less energy is required to photoactivate the photocatalysts for surface radicals generation. 

## 4. Materials and Methods 

### 4.1. Catalysts Preparation

P25 (Aeroxide^®^, average primary particle size 21 nm) was purchased from Sigma-Aldrich, St. Louis, MO, USA. Glycine (henceforth Gly, Acros organics, Morris Plains, NJ, USA) and polytetrafluoroethylene (PTFE, Sigma-Aldrich, St. Louis, MO, USA) were used as sources of nitrogen and fluorine respectively without any further purification. To obtain doped TiO_2_, a four-step process was followed. The first step is the suspension preparation from the as-received powder batches using a suitable dispersant and the titania powder.

In the case of nitrogen doping (N-doping), a 5 wt % aqueous solution of Gly was prepared by dissolving 2.5 g Gly in 500 g of 10^−3^ N nitric acid (HNO_3_) solution. Senna et al. [27] used 100 g in 500 g of the HNO_3_ solution with 5 h of attrition milling. We investigated the effects of milling time and glycine concentration (Supplementary, Section S2), and found a decrease in milling time had little effect and a decrease in glycine concentration showed higher photocatalytic activity (Table S1). For easier processing but maintaining a direct comparison with the previously published work [27], we chose 2.5 g glycine in 500 g of the HNO_3_ solution as this gave the same time to complete bacterial deactivation (120 min) (Table S1). The glycine solution was homogenised for 20 min using an ultrasonic bath. The solution pH was 3.4. 

For fluorine, a 1.92 wt % aqueous suspension of PTFE was prepared by dispersing 2.0 g of PTFE in 102 g of 10^−3^ N HNO_3_ solution. Then, 1.1 g TWEEN 20 (Fluka) was added to the as-prepared PTFE to improve the dispersion of PTFE in the aqueous medium.

In the case of fluorine and nitrogen co-doping (F&N), 1 g PTFE and 55 g of 5 wt % Gly solution were mixed together, which corresponded to half of the quantities used in the cases of N and fluorine doping. We added 10^−3^ N HNO_3_ solution until the total mass of the suspension reached 110 g. Then, 1 wt % of TWEEN 20 was added. For homogeneous dispersion, the suspensions were treated by ultrasonic bath (Branson 5510) for 20 min followed by ultrasonic horn treatment (150 W with Telsonic Ultrasonics, model DG-100) for 15 min.

Finally, 42 g of titania nanopowder was added into 110 g Gly solution, and 21 g of the same batch of titania nanopowder was added into 110 g of PTFE suspension or Gly/PTFE suspension.

In a second step, attrition milling was performed on each suspension as previously described using a Netzsch PE075 mill (Netzsch, Selb, Germany), with yttrium-stabilized zirconia beads (2.5 mm) 180 g, at 1500 rpm for 1 h. The attrition-milled samples (henceforth, samples P25-N-Att, P25-F-Att, and P25-F&N-Att) were washed with either Gly suspension or 10^−3^ N HNO_3_ (2 × 50 mL) to recover the maximum of powder, and dried at 60 °C for 96 h using a SalvisLab Thermocenter TC100 oven. Oven dried powders were then crushed with a mortar and a pestle for 2 min manually and heated in air. Thermogravimetric analysis (TGA) was performed to find the appropriate heat treatment temperatures for all of the samples. The heating temperature was set at to 500/600 °C at a rate of 10 °C/min. The TGA samples were held for 1 h at 500/600 °C, and then cooled down at a rate of 10 °C/min. For the bulk powder samples (4–5 g), they were heated at 10 °C/min up to 500 °C (for N-doping) or 600 °C (for F and F&N), held for 1 h, and cooled naturally (henceforth, samples P25-N-HT, P25-F-HT, and P25-F&N-HT). 

### 4.2. Photocatalytic Activity

The photocatalytic antibacterial activity of P25 powders was assessed by adding 1 g/L titania suspension to an Escherichia coli solution (saline buffer solution, SBS, NaCl (8 mg/L) and KCl (0.8 mg/L) to maintain the osmotic pressure of bacterial cells during the experiment) and exposing the mixture to the solar simulated light (310–800 nm, 50 mW/cm^2^). The initial bacterial solution contained ~4.1 × 10^6^ colony-forming units per milliliter (CFU/mL). Samples were taken every 15 min. During the sampling, the stirring was stopped for 2–3 min, and the samples were taken at different depths of the 40 mL solution. A sample of 100 μL of each run was pipetted onto a nutrient agar plate and then spread over the surface of the plate using the standard plate method. Agar plates were incubated lid down at 37 °C for 24 h before colonies were counted. Three independent assays were done for each sample.

### 4.3. Characterisation

Granulometric analyses were performed for the particle size distribution (PSD) with a Mastersizer S (Malvern Instruments Ltd., Worcestershire, UK). For granulometric analysis, suspensions were prepared in the same SBS (NaCl/KCl) used for antibacterial activity trials: 0.01 g of P25 undoped (P25-UN) or modified P25 powders were mixed with 10 mL of the SBS solution to measure the PSD under the same conditions as used for the photocatalytic activity. The suspension was placed in a plastic container with a magnetic stirrer for 5 min prior to measurements. The colloidal surface properties were also evaluated, i.e., zeta potentials for the as-prepared suspension were measured using Zetasizer Nano ZS (Malvern Instruments Ltd., Worcestershire, UK). The zeta potential surface charge was measured by dispersing the particles in water using an ultrasound bath for 15 min and adjusting the pH between pH 7 and 7.1 (HCl/NaOH 1 M). The nitrogen-specific surface area was assessed using the method of Brunauer, Emmett and Teller (the BET model) with a Gemini 2375 (Micromeritics Instrument, Norcross, GA, USA); samples were degassed under flowing nitrogen at 200 °C for 1 h. 

Solid state properties were determined by X-ray diffractometry (XRD; Philips X Pert, Eindhoven, The Netherlands), thermogravimetric analysis (TGA; TGA/SDTA851e, Mettler Toledo, Columbus, OH, USA), reflectance spectroscopy (Varian Cary 1E spectrometer), X-ray photoelectron spectroscopy (XPS; Axis Ultra, Kratos Analytical, Manchester, UK), Fourier transform infrared spectroscopy (FTIR; Spectrum 100 Optica, Perkin Elmer, Bucks, UK), Raman spectroscopy (LabRAM HR, Horiba, Kyoto, Japan). Scanning Electron microscopy (SEM) images were obtained with a SEM Merlin microscope (Carl Zeiss, Jena, Germany).

## 5. Conclusions

In summary, we have successfully prepared nitrogen-doped, fluorinated, and fluorinated and N-doped (F&N) P25 nanoparticles by a scalable wet milling process in the presence of a doping agent (Gly and PTFE) followed by a heat treatment. XRD and Raman spectroscopy confirmed that all of the samples are nanocrystalline TiO_2_ composed of a mixture of anatase and rutile phases. An increase in the heat treatment temperature from 500 °C to 600 °C for fluorinated samples induced a phase transition with a significant change in anatase to rutile proportions. Heavily agglomerated particles were observed in the saline buffer solution medium, used in the bacterial inactivation evaluation tests. Granulometric analyses and SEM images confirmed a partial sintering after a heat treatment at 600 °C. XPS analysis indicated that N-doping atoms are mainly located in the interstitial position, whereas F-doping atoms are related to a surface fluorination and highlighted the coexistence of a Ti^3+^/Ti^4+^ redox couple. Moreover, FTIR spectroscopy showed higher surface adsorbed hydroxyl groups, which are necessary for photocatalysis applications. All of the doped P25 powders showed effective photocatalytic activity against *E. coli* leading to bacterial inactivation under simulated light. The characterization data seem to indicate that the inactivation process is neither size nor composition-dependent in this case. Among all of the doped samples, results revealed that P25-fluorinated and N-doped nanopowder is the most efficient with a synergistic effect of both N and F non-metal atoms. 

## Figures and Tables

**Table 1:** Summary of the Zeta (ζ) potential measurements of the different P25 samples after attrition milling and after heat treatment at pH 7 in saline buffer solution and the granulometric results and calculated agglomeration factor F_ag_ for the undoped and doped samples. BET: Brunauer, Emmett, and Teller method.

Medium	Sample			Malvern		BET		F_ag_
		pH	ζ Potential [mV]	D_V_50 [μm]	Span	SSA [m^2^.g^−1^]	d_BET_ [nm]	
10^−3^ N HNO_3_ Solution	P25-UN	2	-	2.43	1.72	39.7	35	69
Saline buffer solution	P25-UN	7	−5.1	3.28	2.17	39.7	35	94
	P25-N-Att	7	−9.9	-	-	-	-	-
	P25-N-HT	7	−15.1	20.33	4.01	49.5	28	726
	P25-F-Att	7	−5.3	-	-	-	-	-
	P25-F-HT	7	−21.9	15.19	4.51	23.3	60	253
	P25-F&N-Att	7	−5.9	-	-	-	-	-
	P25-F&N-HT	7	−20.6	21.03	4.49	20.0	70	300

## Supplementary Materials

The following are available online at http://www.mdpi.com/2079-4991/7/11/391/s1, Figure S1: TGA profiles showing the weight loss versus the temperature of P25-UN (a); P25-N-Att (b); P25-F-Att (c) and P25-N&F-Att (d), Figure S2: XPS spectra of undoped and doped samples and high-resolution analysis in the O 1s, Ti 2p, N 1s and F 1s binding energy region, Figure S3: Raman spectra of (a) P25-UN, (b) P25-N-HT, (c) P25-F-HT and (d) P25-F&N-HT. The bands corresponding to rutile were annotated with the letter R, Figure S4: UV-vis-RS spectra of various samples. P25-UN (a) and P25-N-HT (b) (these were separated in the inset for more clarity), P25-F-HT (c) and P25-N&F-HT (d), Figure S5: Bacterial deactivation of E-coli under simulated solar light and in the dark in the presence of N-doped P25 TiO_2_ powder, Table S1: Effect of milling time and glycine concentration on the time to total deactivation of E-coli under illumination of simulated solar light.
