# Supplementary material for: Synergistic Effect of Fluorinated and N Doped TiO2 Nanoparticles Leading to Different Microstructure and Enhanced Photocatalytic Bacterial Inactivation
Source: Nanomaterials (Basel). 2017 Nov 15;7(11):391. doi: 10.3390/nano7110391 (PMC5707608; doi:10.3390/nano7110391)
Supplement: Supplementary file 1 [file nanomaterials-07-00391-s001.pdf]

Support Information

Synergistic Effect of Fluorinated and N Doped TiO_2 Nanoparticles Leading to Different Microstructure and Enhanced Photocatalytic Bacterial Inactivation

Irena Milosevic [1,*], Amarnath Jayaprakash [1], Brigitte Greenwood [1], Birgit van Driel [1,†], Sami Rtimi [1,2] and Paul Bowen [1]

[1] Ecole Polytechnique Fédérale de Lausanne, EPFL-STI-IMX-LTP, Station 12, CH-1015 Lausanne, Switzerland; amarnath.jayaprakash@epfl.ch (A.J.); brigitte.greenwood@gmail.com (B.G.); b.a.vandriel@tudelft.nl (B.v.D.); sami.rtimi@epfl.ch (S.R.); paul.bowen@epfl.ch (P.B.)

[2] Ecole Polytechnique Fédérale de Lausanne, EPFL-SB-ISIC-GPAO, Station 6, CH-1015 Lausanne, Switzerland

* Correspondence: irena.markovic@epfl.ch; Tel.: +41-21-69-35107

† Current address: Materials for Arts and Archeology, 3ME, TU Delft, Mekelweg 2, 2628 CD Delft, The Netherlands

S1. Characterization

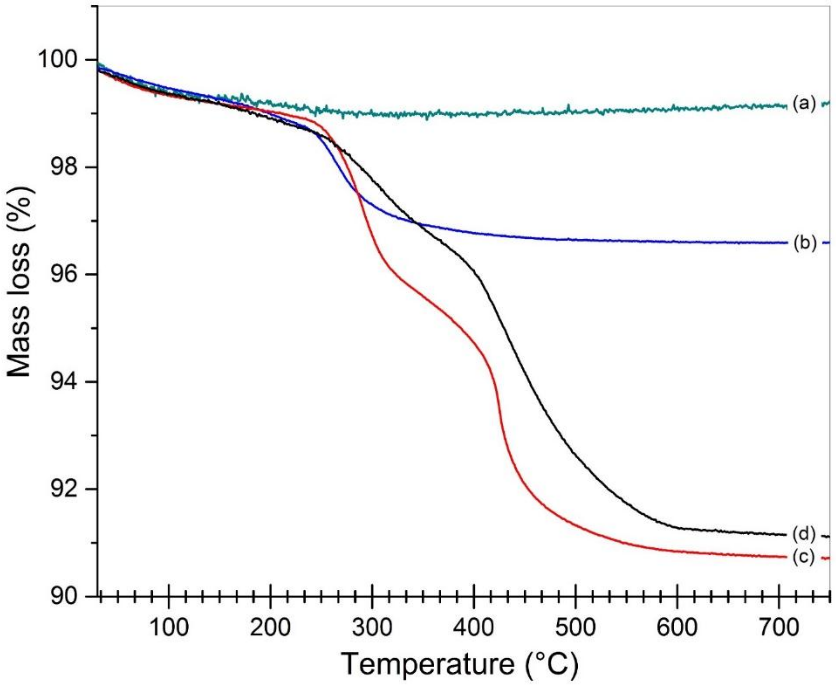

Figure S1. TGA profiles showing the weight loss versus the temperature of P25-UN (**a**); P25-N-Att **(b)**; P25-F-Att (**c**); and P25-N&F-Att (**d**).

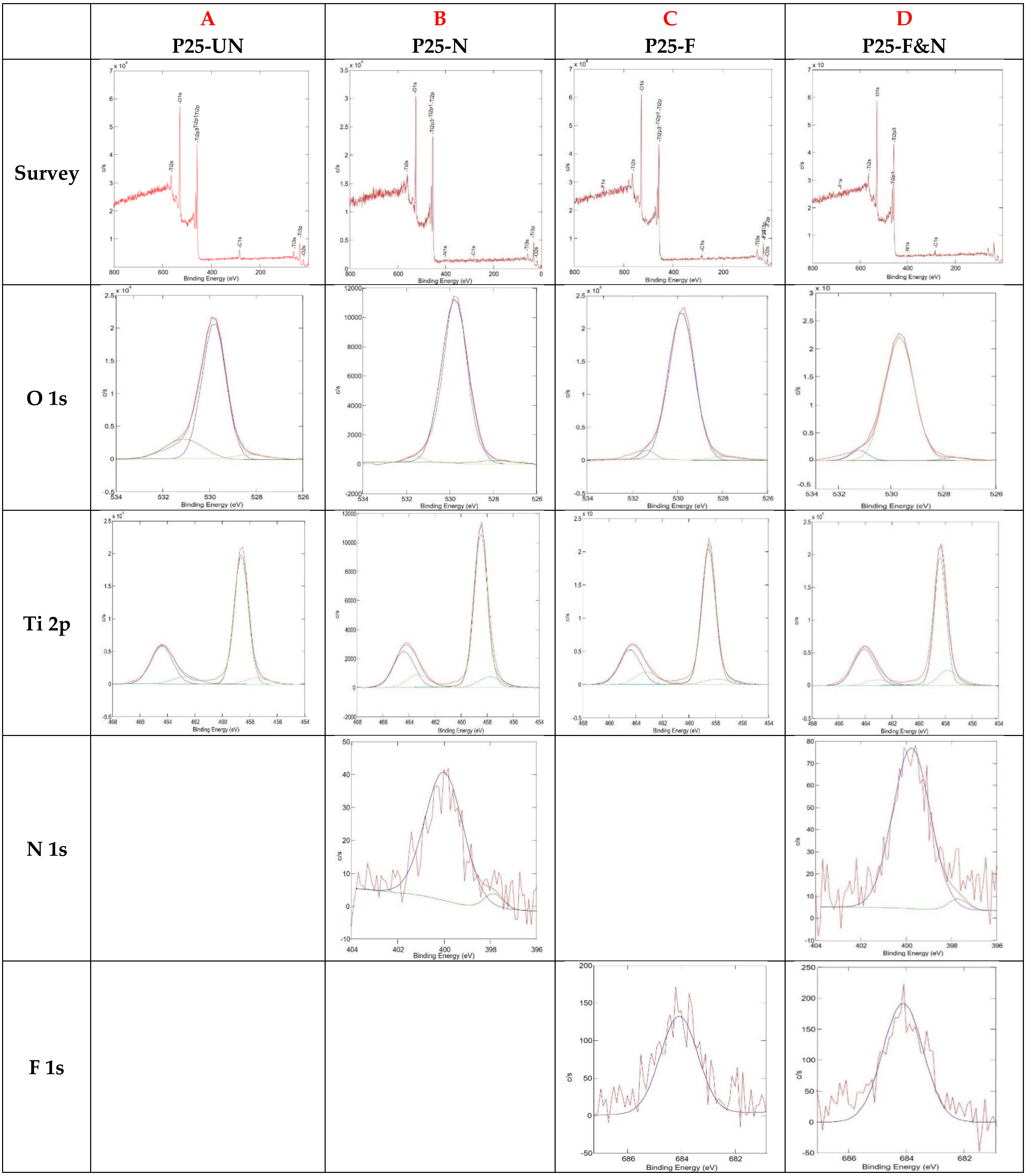

Figure S2. XPS spectra of undoped and doped samples and high-resolution analysis in the O 1s, Ti 2p, N 1s, and F 1s binding energy regions.

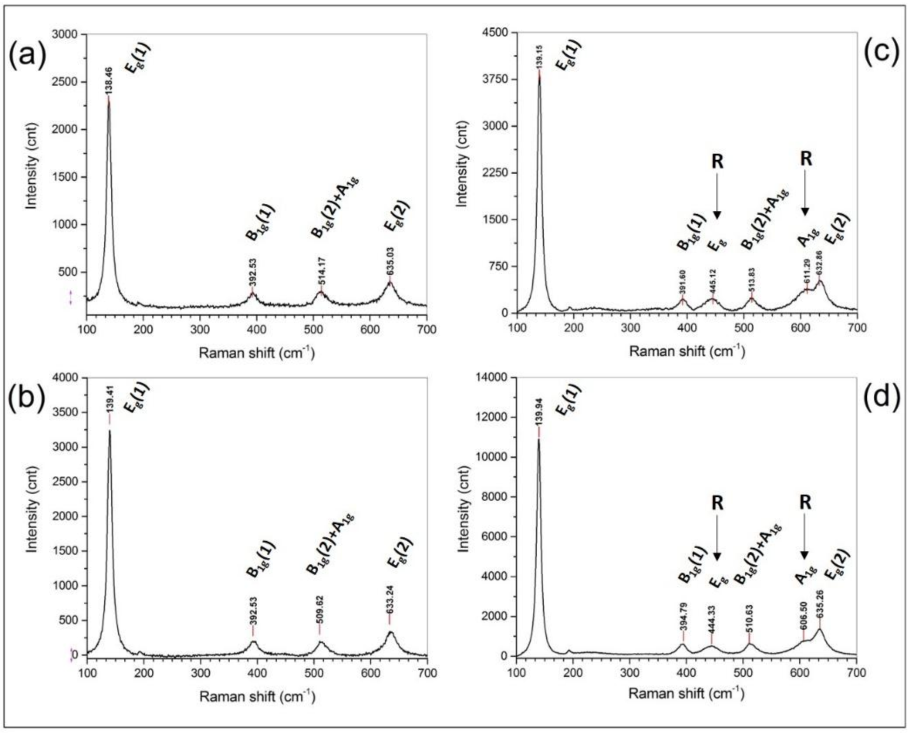

Figure S3. Raman spectra of (**a**) P25-UN; (**b**) P25-N-HT; (**c**) P25-F-HT; and (**d**) P25-F&N-HT. The bands corresponding to rutile were annotated with the letter R.

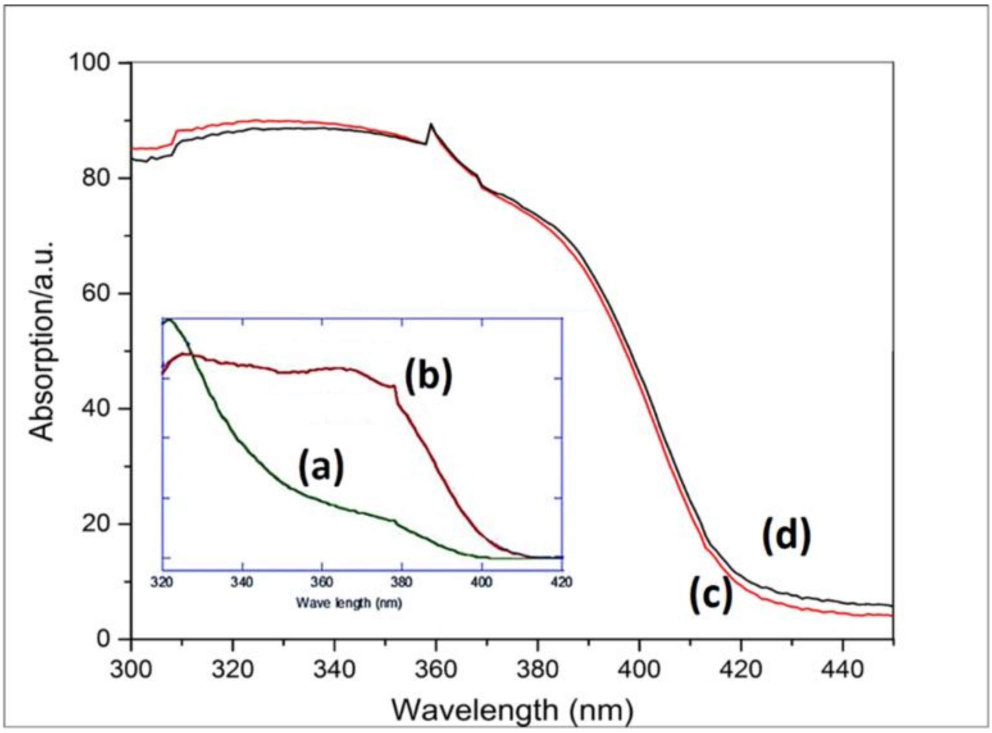

Figure S4. UV-Vis-RS spectra of various samples. P25-UN (**a**) and P25-N-HT (**b**) (these were separated in the inset for more clarity), P25-F-HT (**c**) and P25-N&F-HT (**d**).

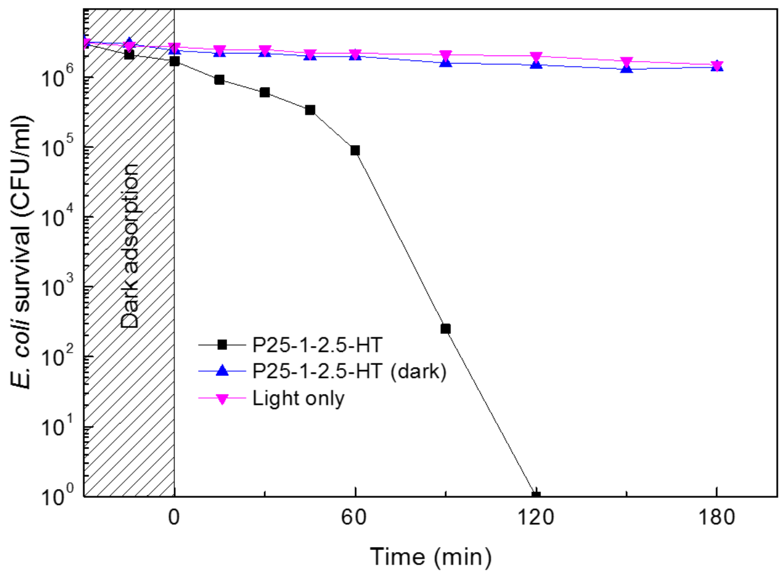

Figure S5. Bacterial deactivation of E-coli under simulated solar light and in the dark in the presence of N-doped P25 TiO_2 powder.

S2. Effect of milling time and Glycine concentration.

For the attrition milling of P25 (see main text for characteristics), different concentrations of glycine ranging from 100g to 2.5 g per 500g of HNO_3 (0.001M) and milling times were investigated with respect to the time to complete the deactivation of E-coli under illumination under solar simulated light (again, as described in the main text). The different tests and results of the photocatalytic activity for the time to total elimination are presented in Table S1. After milling, all of the samples were oven dried and heat treated (500°C for 1 h), as described in the main text.

Table S1. Effect of milling time and glycine concentration on the time to total deactivation of E-coli under illumination of simulated solar light.

Sample	Glycine (g)*	Attrition milling time (h)	Time to complete bacterial deactivation (min)
P25-5-100-HT	100	5	150
P25-3-100-HT	100	3	150
P25-3-25-HT	25	3	90
P25-1-25-HT	25	1	90
P25-1-10-HT	10	1	60
P25-1-5-HT	5	1	180
P25-1-2.5-HT	2.5	1	120

*Amount of glycine in g added to 500g of HNO_3 (0.001M) solution.

We see that as both milling time and glycine concentration decreases to 1 h and 10g glycine (in 500g HNO_3 (0.001 M)), the time to complete deactivation decreases, i.e. the photocatalytic activity increases. For the lowest amount of glycine added (2.5g) with 1 h attrition milling, we get the same time to deactivation as demonstrated by Senna et al. in the original work of N-doping TiO_2 by use of attrition milling and heat treatment [1]. This was chosen for the current studies so that the results of fluorine doping and co-doping are directly comparable with the previously published data.
